# CITED1 confers stemness to Wilms tumor and enhances tumorigenic responses when enriched in the nucleus

**DOI:** 10.18632/oncotarget.1566

**Published:** 2013-12-28

**Authors:** Andrew J. Murphy, Janene Pierce, Christian de Caestecker, Gregory D. Ayers, Alex Zhao, Hernan Correa, Jonathan R. Krebs, Vincente Kenyi Saito-Diaz, Ethan Lee, Alan O. Perantoni, Mark P. de Caestecker, Harold N. Lovvorn

**Affiliations:** ^1^ Department of Pediatric Surgery, Monroe Carell Jr. Children's Hospital at Vanderbilt, Nashville, Tennessee; ^2^ Department of Biostatistics, Vanderbilt University Medical Center, Nashville, Tennessee; ^3^ Department of Cell and Developmental Biology, Vanderbilt University Medical Center, Nashville, Tennessee; ^4^ Cancer and Developmental Biology Laboratory, Center for Cancer Research, National Cancer Institute, Frederick, Maryland; ^5^ Departments of Medicine and Cell and Developmental Biology, Vanderbilt University Medical Center, Nashville, Tennessee; ^6^ Department of Pathology, Vanderbilt University School of Medicine, Nashville, Tennessee

**Keywords:** CITED1, Wilms tumor, WNT, WiT49, cancer stem cell

## Abstract

Wilms tumor (WT) is the most common childhood kidney cancer and retains gene expression profiles reminiscent of the embryonic kidney. We have shown previously that CITED1, a transcriptional regulator that labels the self-renewing, multipotent nephron progenitor population of the developing kidney, is robustly expressed across all major WT disease and patient characteristics. In this malignant context, CITED1 becomes enriched in the nucleus, which deviates from its cytosolic predominance in embryonic nephron progenitors. We designed the current studies to test the functional and mechanistic effects of differential CITED1 subcellular localization on WT behavior. To mimic its subcellular distribution observed in clinical WT specimens, CITED1 was misexpressed ectopically in the human WT cell line, WiT49, as either a wild-type (predominantly cytosolic) or a mutant, but transcriptionally active, protein (two point mutations in its nuclear export signal, CITED1ΔNES; nuclear-enriched). *In vitro* analyses showed that CITED1ΔNES enhanced WiT49 proliferation and colony formation in soft agar relative to wild-type CITED1 and empty vector controls. The nuclear-enriched CITED1ΔNES cell line showed the greatest tumor volumes after xenotransplantation into immunodeficient mice (n=15 animals per cell line). To elucidate CITED1 gene targets in this model, microarray profiling showed that wildtype CITED1 foremost upregulated *LGR5* (stem cell marker), repressed *CDH6* (early marker of epithelial commitment of nephron progenitors), and altered expression of specific WNT pathway participants. In summary, forced nuclear enrichment of CITED1 in a human WT cell line appears to enhance tumorigenicity, whereas ectopic cytosolic expression confers stem-like properties and an embryonic phenotype, analogous to the developmental context.

## INTRODUCTION

Wilms tumor (WT) is the most common childhood kidney cancer and is thought to arise from aberrant mesenchymal to epithelial transition of nephrogenic stem cells [1, 2]. Supporting this postulate, WTs harbor cellular elements that appear reminiscent of the embryonic kidney, including blastema (the malignant analogue of the metanephric mesenchyme and putative cancer stem cell), epithelia (in the form of primitive tubules and glomerular-like structures), and stroma [3, 4]. Moreover, WTs express gene signatures that are retained from the embryonic kidney, yet the functional significance of these developmental pathways, which often designate self-renewing nephron progenitors, is unclear in the malignant context of WT, although fueling perpetuity of its cancer stem cell is one candidate mechanism [5, 6]. Because of these similarities in histology and gene expression profiles between the embryonic and malignant contexts, WTs offer a unique and ideal paradigm to study mechanisms that regulate cancer stem cell self-renewal and epithelial conversion.

CITED1 is a non-DNA binding transcriptional co-activator that labels the multipotent population of self-renewing nephrogenic stem cells in the cap mesenchyme (CM) of the embryonic kidney [7, 8]. Upon the earliest commitment to epithelial transition of this nephron progenitor pool, CITED1 is repressed, and its overexpression in cultured metanephric mesenchymes (MM) blocks epithelial differentiation [9]. Furthermore, CITED1 is no longer detected in the maturing kidney once the CM has dissipated and nephrogenesis has been completed, and is absent in the normal adult kidney. However, from our earlier studies, CITED1 is robustly detected in WTs of all major disease and patient characteristics, which makes it a potentially attractive and unifying therapeutic target [7, 10, 11]. One fundamental observation from these previous studies is that CITED1, which in the CM of the developing kidney is predominantly detected in the cytosol, becomes enriched in the nuclei of WT blastema [7, 10]. Control of CITED1 subcellular localization is currently unknown, although it is developmentally regulated, showing cytosolic predominance in nephron progenitors and hepatic primordia, but a nuclear preference in trabeculae of the developing heart [12]. Diverging further from its expression domain in the embryonic kidney, in which it is absent among the earliest nephronic structures, CITED1 can be detected rarely in primitive epithelial structures of WT but appears principally to be a marker of blastema. Mechanistically, we have shown that mutation of the transcriptionally active carboxy terminus of CITED1 perturbs in a dominant-negative manner embryonal tumor cell proliferation *in vitro* and tumorigenesis in a xenograft model [13]. However, we have yet to clarify the molecular pathways and targets of CITED1 that promote Wilms tumorigenesis and that control its subcellular trafficking, and until now, whether differential CITED1 subcellular localization between the embryonic and malignant contexts confers any functional significance. Depending on its subcellular localization, CITED1 may impart dual functions that cooperatively benefit the multiple survival pathways of a cancerous cell, by conferring stemness when predominantly cytosolic, analogous to the embryonic state, and by enhancing tumorigenicity when enriched in the nucleus.

As a clue to these functional mysteries of CITED1 in WT, our efforts to uncover its interactions in the developing kidney have shown that this transcriptional co-activator is a repressor of canonical WNT/β-catenin signaling and blocks epithelial differentiation of nephron progenitors [9]. Indeed, perturbations in WNT/β-catenin signaling have been linked to Wilms tumorigenesis in humans and in animal models [14–17]. Although deregulated activation of WNT/β-catenin signaling is thought to be oncogenic, integrity of this pathway in the developing kidney is necessary for proper nephrogenesis to proceed, possibly by maintaining stemness of the nephron progenitor pool and also promoting nephron progenitor expansion [18–21]. Although WNT/β-catenin plays a critical role in proper nephrogenesis, induction of epithelial differentiation appears to be more dependent on non-canonical WNT signaling mechanisms [22]. Progenitors residing in an embryonic niche that are required to stay in a stem state must resist differentiation signals by maintaining specific counterbalance mechanisms. Mechanisms that inhibit differentiation of the progenitor cell pool and thereby maintain stemness may be at the core of stem cell perpetuity, either in the normal developing kidney or in the malignant context of WT.

These current studies were designed to test the hypotheses that predominantly cytosolic CITED1 mimics the embryonic context and confers stemness to WT through WNT inhibition and that CITED1 nuclear enrichment enhances Wilms tumorigenic responses. This study therefore seeks to clarify the functional significance of our prior observations that CITED1 subcellular localization is perturbed in the malignant context, becoming nuclear enriched.

## RESULTS

### CITED1 subcellular localization in experimental model of WT

**Figure 1:**
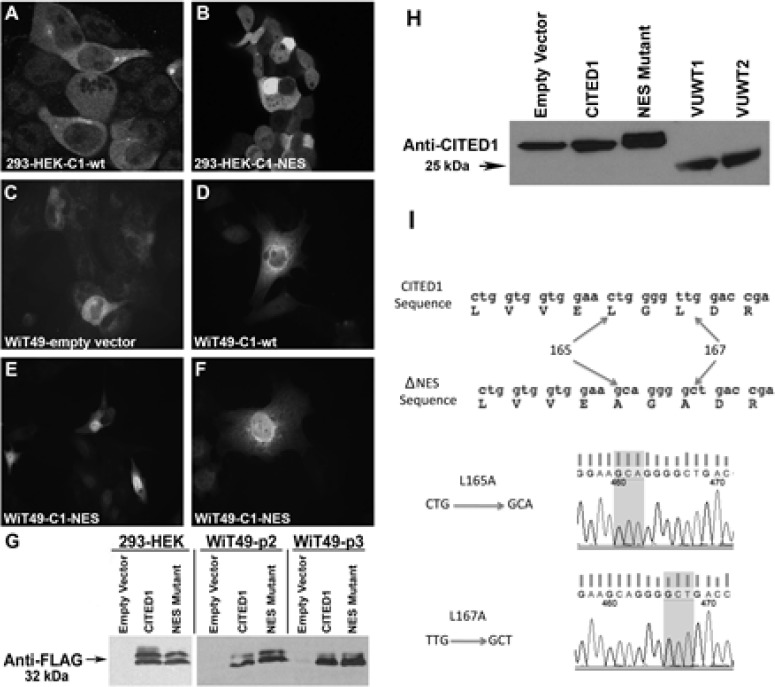
Validation of transgene expression in cultured 293-HEK and WiT49 cells *(A)* Transfection of wild-type CITED1 (C1-wt) in 293-HEK cells shows predominantly cytosolic immunofluorescent (IF) detection. *(B)* Transfection of CITED1ΔNES (C1-NES) shows enriched nuclear IF detection. *(C)* WiT49 cells transfected with empty vector control plasmid show rare and weak cytosolic CITED1 IF detection. *(D)* Wild-type CITED1 overexpression shows predominantly cytosolic IF detection. *(E, F)* CITED1ΔNES transfection into WiT49 cells shows robust nuclear and weak cytosolic IF detection. *(G)* Western blot for the FLAG epitope in 293-HEK and WiT49 cells transfected with experimental transgenes (p, passage number). *(H)* Western blot for FLAG-tagged CITED1 in WiT49 cells and two Wilms tumor specimens (VUWT1 and 2). *(I)* Direct sequencing confirms L165A and L167A mutations in the nuclear export signal (nuclear-preference) of the CITED1ΔNES plasmid.

To mimic its differential subcellular localization observed between embryonic kidney and clinical WT, wild-type and mutant CITED1 (ΔNES) were expressed ectopically in WiT49 cells. For comparison in the embryonic context, these constructs were also transfected into 293-HEK cells. As expected in both cell lines, wild-type CITED1 showed predominantly cytosolic localization, whereas CITED1ΔNES was enriched robustly in the nucleus (Figure 1, A–F). Western blot showed detection of the FLAG epitope and CITED1, confirming integrity of transgene expression (Figure 1, G–H). The point mutations responsible for two desired amino acid substitutions in the NES domain (L165A and L167A) were confirmed by direct sequencing (Figure 1I).

### Effects of CITED1 misexpression on Wilms tumorigenesis

#### In vitro

**Figure 2:**
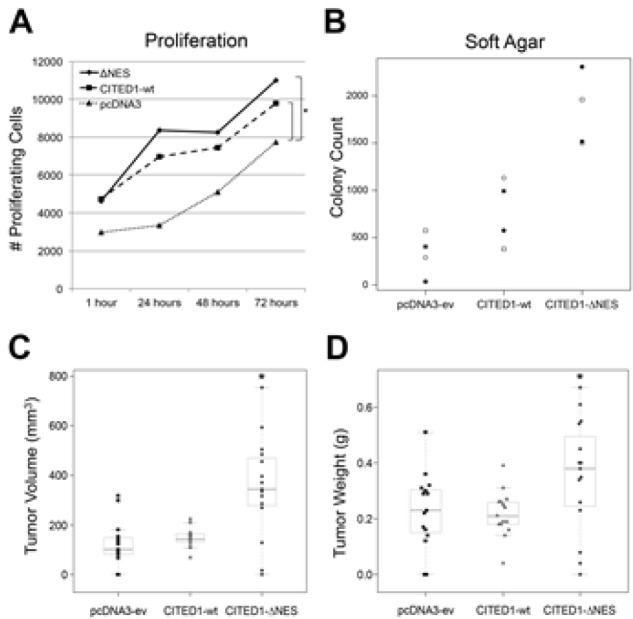
Functional effects of CITED1 misexpression in WiT49 cells *(A)* Relative to empty vector WiT49 control cells, overexpression of both CITED1ΔNES and wild-type CITED1 enhanced proliferation at each time point tested (*p >0.001), although the rate of change over time did not vary (p=0.29), and no significant difference was observed between CITED1ΔNES and wild-type CITED1. *(B)* When the three WiT49 cell lines were grown in soft agar (four biological replicates; open/closed circles and squares correspond to each experiment), CITED1ΔNES significantly enhanced colony formation relative to wild-type CITED1 (2.22 fold, p=0.0286) and to empty vector controls (5.01 fold, p=0.0286). Wild-type CITED1 colony count was not statistically different from empty vector control (p=0.2). *(C)* When xenotransplanted into immunodeficient mice, each WiT49 cell line showed similar growth characteristics as in soft agar. CITED1ΔNES tumors showed the highest mean tumor volumes relative to the other cell lines (p >0.0001; all data points shown). *(D)* Mean tumor weights were also different between the three cell lines (p=0.028) but showed greater variability. CITED1ΔNES weights were significantly greater than wild-type CITED1, although differences between wild-type and empty vector were not statistically different.

WiT49 cells transfected with either wild-type CITED1 or ΔNES vector showed a greater number of proliferative cells than with pcDNA3 empty vector at all time points (Figure 2A; p >0.001), but the rate of change in the number of proliferative cells between each time point was not statistically different for the wild-type and CITED1ΔNES cell lines (p=0.29). These data imply that CITED1 enhances the baseline proliferative response of WiT49 cells and that this difference remains relatively constant over time.

When grown in soft agar, WiT49 cells expressing CITED1ΔNES showed the highest normalized colony counts, followed by cells expressing wild-type CITED1 (Figure 2B). The median normalized colony count for ΔNES was 5.01 times greater than empty vector control cells (p=0.0286) and 2.22 times greater than wildtype CITED1 expressing cells (p=0.0286). The median normalized colony count for wild-type CITED1 cells was not statistically significant when compared to empty vector control cells (p=0.2).

#### In vitro

After xenotransplantation into immunodeficient mice, statistically significant growth differences were observed between the three experimental WiT49 cell lines when measuring tumor volumes (mm^3^; Figure 2C; p >0.0001) and tumor weights (grams; Figure 2D; p=0.028); however, no difference was observed in tumor incidences between cell lines (empty vector: 13/15; CITED1: 15/15; ΔNES: 14/15). For tumor volume, the minimum statistically significant difference between groups was 115 mm^3^ (p >0.0001). CITED1ΔNES tumor volumes (mean = 350.6 ± 202.3) were statistically greater than volumes for both wild-type CITED1 (mean = 147.9 ± 38.1) and empty vector controls (mean = 124.0 ± 89.9; Figure 2C; p >0.0001), although statistical significance was not achieved for the differences observed between these latter two groups. For tumor weight, the minimum statistically significant difference between groups was 0.132 grams (p=0.028). CITED1ΔNES tumor weights (mean = 0.35 ± 0.2) were statistically greater than tumor weights for wild-type CITED1 (mean = 0.22 ± 0.08; p >0.05), although empty vector tumor weights (mean = 0.23 ± 0.14) did not differ statistically from either CITED1 group (Figure 2D).

### Experimental WiT49 tumor histology

**Figure 3:**
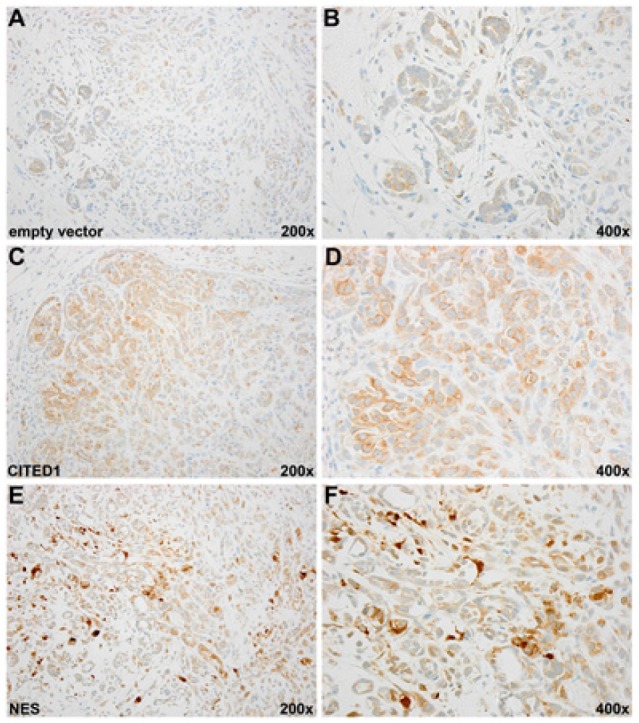
(A, B) Low and high magnification images of an empty vector WiT49 tumor immunostained for CITED1. Although no histologic alterations in cellular composition were observed between the three cell lines, lower cell density and fewer cells with nuclear atypia were qualitatively found in empty vector tumors relative to the two CITED1 overexpressing cell lines. Immunostaining for CITED1 showed the expected weak cytosolic detection typical of parent unmanipulated WiT49 cells (data not shown). *(C, D)* Immunoperoxidase staining for CITED1 was increased predominatly in the cytosol of wild-type CITED1 tumor with occasional nuclear enrichment (low and high magnification images shown). *(E, F)* CITED1ΔNES tumor shows the expected nuclear enrichment of CITED1 with weaker cytosolic detection, findings that mimic the expression domain of clinical WT specimens. Notably, cells with nuclear atypia were more prevalent in CITED1ΔNES tumors.

As expected, immunohistochemical detection of CITED1 in empty vector WiT49 tumors was weaker and predominantly cytosolic, particularly in primitive epithelial elements analogous to parent WiT49 tumors (Figure 3 A, B). Experimental tumors expressing wild-type CITED1 showed more intense CITED1 detection, which again was predominantly cytosolic, although rare cells expressed nuclear CITED1 (Figure 3 C, D). CITED1ΔNES tumors showed robust nuclear expression of CITED1 and frequently concomitant cytosolic detection, analogous to its expression domain in clinical WT (Figure 3 E, F). Nuclear atypia (large, hyperchromatic nuclei) appeared particularly prevalent in these CITED1ΔNES cellular compartments.

### Gene expression analysis (Table 1)

Given these functional differences between cytosolic and nuclear enriched CITED1, we conducted a gene expression analysis in each of the three experimental cell lines (n = 3 biological replicates per line), using the empty vector control line as the reference cells, to clarify the effects of ectopic CITED1 expression on the gene profiling of WiT49 cells. In a microarray screen of over 36,000 gene transcripts, we detected several interesting patterns of up or down regulated genes (Table 1). When comparing gene expression profiles between wild-type CITED1 and empty vector controls, the single most upregulated gene was *LGR5* (Leucine-rich repeat-containing G-protein coupled receptor 5), and the single most downregulated gene was *CDH6* (cadherin-6), suggesting that cytosolic CITED1 may confer a stem-like phenotype to WT. Alterations in several other developmentally regulated genes fit this pattern of cytosolic-enriched CITED1 bestowing an embryonic phenotype to WT. Specifically, *LIN7A* (lin7-homologue A; cell adhesion molecule that interacts with membrane-bound β-catenin) was the 3^rd^ most repressed gene, and *HOXA2* (homeobox protein A2; represses the transcription factor, *SIX2*, which facilitates self-renewal and maintenance of nephron progenitors) was the 4^th^ most downregulated gene. *DKK1* (dickkopf-1; secreted WNT antagonist) was the 4^th^ most upregulated gene, and *PTGS2* (prostaglandin-endoperoxide synthase-2; protein product is COX2) was the 7^th^ most upregulated gene. When comparing gene expression profiles between CITED1ΔNES and empty vector controls, the most striking observation was that among ΔNES cells the single most upregulated gene was CITED1. Notably, late passages (>12) of WiT49 cells ectopically expressing CITED1ΔNES show increasing levels of CITED1 protein on Western blot (data not shown), which was one reason for restricting all analyses to passages earlier than 9.

**Table 1:** Results from gene expression analysis of WiT49 cells after CITED1 misexpression Comparisons were made between three biologic replicates of cells expressing either wild-type CITED1 or ΔNES CITED1 and empty vector controls. Genes are displayed in rank order of fold change, either up- or down-regulated. Known biological functions for each gene are included.

Cell Line: versus empty vector	Gene	Fold Change	Rank Order	Biological Function
	*LGR5*	+ 2.04	1	Stem cell marker; direct target of Wnt/CTNNB1
	*PLAC8*	+ 1.53	2	Onzin (synonym); enhances proliferation; pro-survival; retinoids repress, inducing differentiation
	*NT5E*	+ 1.52	3	CD73 (synonym); target of Wnt/CTNNB1; associated with cancer progression
	*DKK1*	+ 1.48	4	Wnt inhibitor; represses epithelial commitment of nephron progenitors; promotes stemness
	*GREM1*	+ 1.48	5	Wnt/CTNNB1 target; BMP antagonist; pro-stem effects
CITED1	*CD34*	+ 1.45	6	Marker of hematopoietic and endothelial stem cells
	*PTGS2*	+ 1.40	7	COX2 (synonym); synthesizes prostaglandins (PGE2); important to normal nephrogenesis; regulator of tumorigenesis and disease progression; anti-apoptosis effects; Wnt1/CTNNB1 target
	*PLA2G4A*	− 1.50	5	Phospholipase (A)2; inactivating mutations enhance tumor progression
	*RNU13P2*	− 1.51	4	SNORD13P2; no reported function
	*HOXA2*	− 1.51	3	Transcription factor that represses SIX2 (developmentally regulated gene)
	*LIN7A*	− 1.52	2	Recruits cell adhesion molecules to interact with β -catenin; stabilizes cytoskeleton through β-catenin
	*CDH6*	− 1.62	1	Early marker of epithelial commitment of nephron progenitors; labels pretubular aggregate of embryonic kidney
	*CITED1*	+ 1.96	1	Marker of nephron progenitors in embryonic kidney; proproliferative effects in malignant context
	*KIR2DS2*	+ 1.62	2	Killer cell immunoglobulin-like receptors (KIRs); transmembrane glycoproteins
	*LCN1L1*	+ 1.54	3	Lipocalin-1-pseudogene-1; function not reported
ΔNES	*TUBA3E*	+ 1.50	4	Tubulin; component of microtubules, which form the spindle fibers for separating chromosomes during mitosis
	*BTBD11*	− 1.50	3	All-trans retinoic acid responsive gene; induces differentiation
	*SNORD56B*	− 1.57	2	No reported function
	*RNU13P2*	− 1.58	1	SNORD13P2; no reported function

### WNT pathway analysis (Table 2)

We have shown previously that CITED1 repressed epithelial differentiation of cultured metanephric mesenchymes, and induced the WNT inhibitor *KREMEN1*, the DKK1 receptor, in a hepatoblastoma cell line [9, 12]. Given that ectopic expression of wild-type CITED1 induced *DKK1* in WiT49 cells from the above microarray, we sought to validate these observed changes and to screen for additional alterations in known effectors of the WNT signaling cascade using this experimental model. Interestingly, in WiT49 cells expressing ectopic wildtype CITED1, a pattern of WNT antagonism appeared at multiple pathway entry points but most notably through induction of *DKK1* and its receptor, *KREMEN1*. Of the 18 WNT pathway genes that were up or down regulated in this wild-type CITED1 experimental cell line, a logical relationship emerged that wild-type CITED1 is promoting stemness through inhibition or stimulation of key regulators of stem cell maintenance (Table 2). Upon ectopic expression of CITED1ΔNES (nuclear CITED1), five WNT pathway genes were affected, showing a pattern consistent with CTNNB1 stabilization. For example, *CTNNBIP1* is known to displace CTNNB1 from the TCF/LEF complex and thereby inhibits target gene transcription. However, when repressed, this effect is potentially permissive for CTNNB1 to exert its oncogenic properties.

**Table 2:** Results from WNT pathway and Cancer Drug Target real time PCR arrays Genes are displayed having fold changes either up- or down-regulated and a p-value >0.05. Known biological functions for each gene are included.

WNT	Gene	Fold change	p-value	Biological Function
	*CCND2*	−3.0	0.0004	β-catenin target gene; facilitates cell cycle progression from G1 to S phases
	*WNT2B*	−4.6	0.0004	Induces nephrogenesis; promotes epithelial differentiation
	*FZD4*	−1.9	0.0039	Promotes ureteric bud branching morphogenesis
	*DAAM1*	−1.4	0.0075	Maintains cytoskeleton architecture; knockdown reduces pronephric epithelial markers
	*FRZB*	−2.7	0.0089	sFRP3; candidate tumor suppressor
	*WNT11*	−1.4	0.0093	Secreted from UB tips; regulates UB epithelial branching; non-canonical pathway; redundant function with WNT5B
	*SLC9A3R1*	−1.6	0.0117	Scaffold protein; may enhance Wnt signaling
	*PITX2*	−1.6	0.0152	Morphogen
CITED1	*KREMEN1*	+1.6	0.0278	DKK1 receptor; Wnt antagonist
	*NLK*	−1.2	0.0295	Serine/threonine kinase; negative regulator of Wnt/β-catenin nuclear effects (analogous to CTNNBIP1)
	*WNT5B*	−1.7	0.0341	Non-canonical; detected in minority of Wilms tumor; redundant function with WNT11
	*TCF7L1*	−1.3	0.0372	Mediates Wnt/β-catenin signaling; necessary for terminal differentiation
	*DKK1*	+4.6	0.0379	Wnt inhibitor; represses nephrogenesis; promotes stemness
	*SENP2*	−1.2	0.0428	Sumoylation activity
	*CTNNBIP1*	−1.7	0.0440	Wnt antagonist; promotes ureteric bud branching morphogenesis; down-regulated in 1p-deleted WT
	*CSNK2A1*	−1.4	0.0458	Serine/threonine kinase
	*FBXW2*	−1.5	0.0488	Ubiquitin ligase
	*WNT16*	−1.5	0.0494	Developmentally regulated activity
	*BTRC*	−1.4	0.0065	Ubiquitin ligase; ubiquitinates β-catenin
	*NLK*	−1.2	0.0295	Serine/threonine kinase; negative regulator of Wnt/β-catenin nuclear effects (analogous to CTNNBIP1)
ΔNES	*CTNNBIP1*	−1.7	0.0296	Wnt antagonist; promotes ureteric bud branching morphogenesis; down-regulated in 1p-deleted WT
	*DVL1*	+1.2	0.0339	Signal transducer of Wnt pathway; pro-proliferative; increases β-catenin tumorigenicity in neuroblastoma
	*CCND2*	−1.6	0.0409	β-catenin target gene; facilitates cell cycle progression from G1 to S phases
TARGETS	*Gene*	Fold change	p-value	Biological Function
CITED1	*IGF2*	+1.5	0.017	Loss of imprinting of IGF2 is the most common genetic or epigenetic alteration found in Wilms tumor (~50%)
	*PTGS2*	+2.2	0.026	Synthesizes prostaglandins (PGE2); important to normal nephrogensis; regulator of tumorigenesis and disease progression
	*CTSD*	−2.1	0.037	Stimulates apoptosis
	*PTGS2*	+2.1	0.043	Synthesizes prostaglandins (PGE2); important to normal nephrogensis; regulator of tumorigenesis and disease progression
ΔNES	*HDAC11*	−1.7	0.044	Transcriptional co-repressor; binding partner of HDAC6; expressed specifically in kidney
	*PIK3C2A*	−1.6	0.046	Receptor-linked serine/threonine kinase; AKT pathway
	*AKT1*	−1.8	0.048	Cytosolic serine/threonine kinase

### Cancer drug target analysis (Table 2)

To validate and explore further the gene expression microarray finding that *PTGS2*, the gene that codes for COX2, an enzyme readily inhibited by NSAIDS, is upregulated in WiT49 cells expressing wild-type CITED1, we performed real-time PCR using the Cancer Drug Target array analysis, which includes *PTGS2* and several other genes detected on the microarray screen above. Of a possible 84 cancer drug target genes evaluated in this assay, two were noted to be significantly induced in this experimental cell line, specifically *PTGS2* and *IGF2*. Notably, the CITED1ΔNES line also upregulated *PTGS2*, although its other effects on cancer drug targets were suppressive in this model.

### Canonical WNT reporter assay

**Figure 4:**
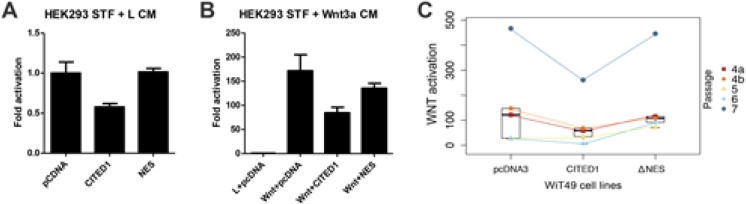
Misexpression of CITED1 both in human embryonic kidney cells (HEK293) that have been stably transfected (STF) with a TOPflash reporter and in WiT49 cells that had subsequent transient transfection of a TOPflash reporter *(A)* Empty vector control (pcDNA) shows baseline TOPflash activation in HEK293 cells bathed in L-cell conditioned medium (L CM). Wild-type CITED1 shows almost two-fold repression of TOPflash activation, whereas transfection of the CITED1ΔNES shows restored responsiveness. *(B)* HEK293 cells stably transfected with a TOPflash plasmid and grown in the presence of Wnt3a CM showed marked reporter assay activation, as estimated from the empty vector controls (pcDNA). Transfection of wild-type CITED1 in these HEK293 cells showed more than two-fold repression of Wnt3a activation, whereas transfection with the CITED1ΔNES overcame much of this repressive function. Experiments for *A* and *B* were completed in triplicate and repeated on consecutive weeks. *(C)* We observed an identical effect of these CITED1 constructs when stably transfected into malignant WiT49 cells and then subsequently transiently transfected with TOPflash. Four biological replicates were performed with different passages (4–7), and one passage was repeated (4a and 4b). Regardless of passage number, overexpression of wild-type CITED1 in WiT49 cells repressed Wnt3a activation, whereas the CITED1ΔNES again appeared to overcome this repressive effect. Notably, in our model, wild-type CITED1 in nearly exclusively cytosolic in both HEK293 and WiT49 cells, but the CITED1ΔNES shows nearly evenly distributed subcellular localization between the cytosol and nucleus, which may reflect a mixed response effect.

To validate that CITED1 modifies canonical WNT signaling in this model, TOP-FLASH luciferase assay (FOP-FLASH control) for β-catenin activity was compared among four passages of pcDNA3 empty vector, CITED1, and CITED1ΔNES cell lines. Overexpression of wild-type CITED1 abrogated TOP-FLASH reporter activity when compared to pcDNA3 empty vector control; however, TOP-FLASH reporter activity was not repressed in the CITED1ΔNES cell line (Figure 4). This effect was demonstrated in both HEK293 and WiT49 cell lines.

### Validation of gene expression and qPCR array analyses in experimental model and clinical WT

#### Experimental model

**Figure 5:**
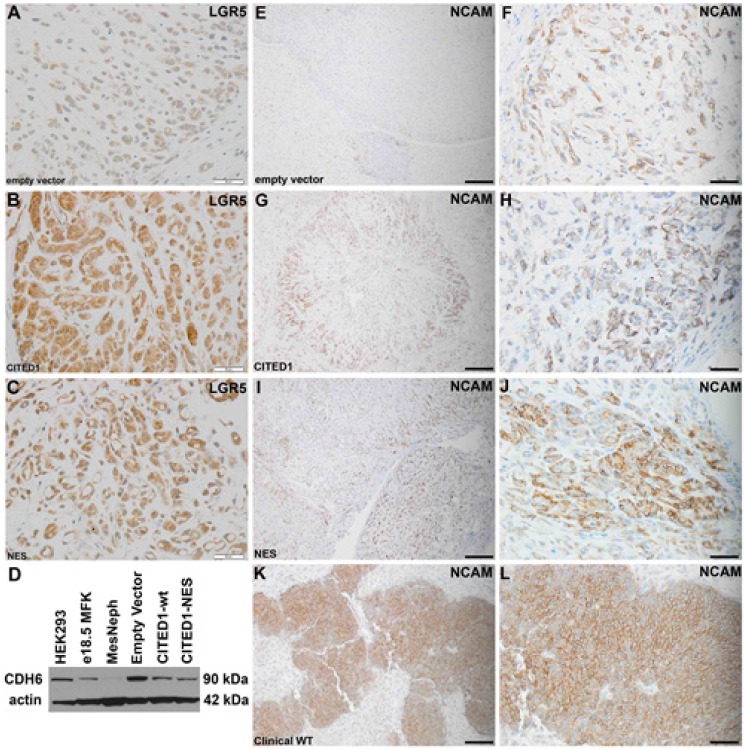
Validation of LGR5 upregulation and CDH6 repression in experimental WT model *(A)* Immunoperoxidase staining for LGR5 (brown) in pcDNA3-empty vector WiT49 xenograft tumors. Detection is weak and cytosolic (400x; bar, 50 μm). LGR5 detection is strongest in pcDNA3-CITED1 WiT49 xenograft tumors *(B)* and intermediate in pcDNA3-CITED1ΔNES WiT49 xenograft tumors (*C*; 400x; bar, 50 μm). *(D)* Western blot for CDH6 protein in cell and tissue lysates. 293-HEK control cells; MFK, mouse fetal kidney homogenate; MesNeph, mesoblastic nephroma control renal tumor (non-WT); three experimental cell lines. Actin, loading control. Relative to the WiT49-empty vector control line, CDH6 is reduced in both wild-type (wt) and mutant (ΔNES) WiT49 cell lines. *(E–F)* Weak immunohistochemical detection of the WT stem cell marker NCAM is present in Wit49 pcDNA3-empty xenograft vector tumors (E; 200X; bar 100 μm; F; 400X; bar 100 μm). *(G–H)* Stronger detection of NCAM is present in Wit49 pcDNA3-CITED1 xenograft tumors (G; 200X; bar 100 μm; H; 400X; bar 100 μm). *(I–J)* Similar detection of NCAM was observed in WiT49 pcDNA3-CITED1ΔNES xenograft tumors (I; 200X; bar 100 μm; J; 400X; bar 100 μm). *(K–L)* Immunohistochemistry for NCAM in a human WT specimen is shown for comparison. (K; 200X; bar 100 μm; L; 400X; bar 100 μm).

To validate the concomitant pro-stem effects of CITED1 to upregulate *LGR5* and downregulate *CDH6* in our WT model, we immunostained LGR5 in xenograft tumors originating from each of the three experimental cell lines. Interestingly, WiT49 tumors from all three lines showed some degree of LGR5 detection (Figure 5, A–C). Empty vector tumors showed weak cytosolic LGR5 detection (Figure 5A), whereas tumors overexpressing wild-type CITED1 showed the greatest LGR5 detection (Figure 5B). CITED1ΔNES tumors showed an intermediate intensity of LGR5 expression (Figure 5C). Western blot for CDH6 from lysates of the three experimental cell lines showed a reduced content in the wild-type CITED1 cells relative to empty vector cells; a similar reduction in CDH6 protein detection was also seen in CITED1ΔNES cells (Figure 5D). To examine alterations in overall differentiation of these experimental tumors, immunostaining for NCAM (neural cell adhesion molecule), a marker of undifferentiated blastema, the putative WT cancer stem cell, was performed to specify an “undifferentiated” phenotype [23, 24]. All tumor lines and specimens expressed NCAM, showing variable content that was histology-dependent and appearing more prevalent in the CITED1 WiT49 cell lines (Figure 5E–L).

#### Clinical WT

**Figure 6:**
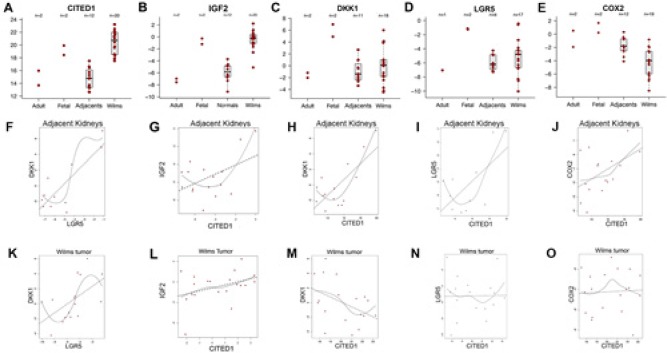
Box plots showing relative mRNA content of four WNT/β-catenin target genes in adult (n=2) and fetal (n=2) kidney specimens (reference values), kidney tissue adjacent to WT (adjacents, n=12), and WT (n=20) *(A)* Median *CITED1* content in WT was 63.4-fold greater in WT than in ipsilateral and adjacent kidney tissue (p >0.0001). Note the approximation of fetal and WT *CITED1* content and adult with adjacent kidneys. *(B) IGF2* content was 55 fold greater in WT specimens when compared to adjacent kidneys (p >0.0001). *(C)* Similar separation in *DKK1* content was observed between reference adult and fetal kidneys. Median *DKK1* content was not statistically different between WT and adjacent kidneys (p=0.418). *(D)* *LGR5* mRNA content showed a similar pattern to DKK1, and was not statistically different between WT and adjacent kidneys (p=0.294). *(E)* PTGS2 mRNA content also overlapped closely between adult and adjacent kidneys, however, its median content in WT was 4.6-fold lower in WT specimens than adjacent kidneys (p >0.001). *(F)* A significant correlation was detected between *LGR5* and *DKK1* mRNA content in adjacent kidneys (ρ=0.82/p=0.002). *(G–J)* CITED1 mRNA content in the non-cancerous kidney tissues showed a consistent trend towards a positive association with each of the other three genes: IGF2 ρ=0.48/p=0.06 *DKK1* ρ=0.74/p=0.002; *LGR5* ρ=0.64/p=0.03; *PTGS2* ρ=0.48/p=0.06; although insufficient observations were present to meet statistical significance in all cases. *(K)* A significant correlation was detected between *LGR5* and *DKK1* mRNA content in WT (ρ=0.56/p=0.03). *(L–O)* In WT, however, this trend towards a positive association between *CITED1* and the other three genes was attenuated, lost, or paradoxically reversed with *DKK1*: *IGF2* ρ=0.37/p=0.10; *DKK1* ρ=-0.42/p=0.08; *LGR5* ρ=0.02/p=0.95; *PTGS2* ρ=0.05/p=0.84.

Clinical WT and adjacent kidney specimens were evaluated using qPCR for the relative mRNA content of the following genes of interest, which are direct or indirect targets of WNT/β-catenin and were up-regulated in our analyses: *CITED1*, *DKK1*, *LGR5*, *PTGS2*, and *IGF2*. The content of these genes of interest in the adult and fetal kidney specimens are displayed for reference, after normalization to *GAPDH* content (Figure 6A–E). As expected, median *CITED1* mRNA content was markedly greater (63-fold) in WT specimens relative to the adjacent kidneys (Figure 6A; p >0.0001), as similarly was *IGF2* content (55-fold; p >0.0001; Figure 6B). Predictably, the *CITED1* and *IGF2* content in pooled fetal kidneys approximated the WT specimens, whereas the adult and adjacent kidney specimens showed similar *CITED1* content. Median *DKK1* and *LGR5* mRNA contents were 2.8- and 2.6-fold greater respectively in WT specimens relative to adjacent kidneys, although an insufficient number of observations likely precluded reaching statistical significance (Figure 6C, p=0.418; Figure 6D, p=0.294). Unexpectedly, median *PTGS2* mRNA content was significantly reduced (4.6-fold) in WTs relative to adjacent kidneys (Figure 6E; p >0.001).

To determine any association between transcription of these five WNT/β-catenin target genes of interest (ie, *CITED1*, *DKK1*, *LGR5, PTGS2*, and *IGF2*), a Spearman correlation coefficient was applied to non-cancerous specimens (ie, adjacent kidneys, and adult and pooled fetal kidneys) and to the WT specimens. Interestingly, a trend towards a positive association (ρ ≥ 4.0) was observed for *CITED1* and each of the other four genes of interest in the non-cancerous kidney tissues (Figure 6 G–J), but curiously this potential relationship was lost in the WT specimens (Figure 6 L–O). A statistically significant correlation was observed between DKK1 and LGR5 both in adjacent kidneys (Figure 6F) and in WT specimens (Figure 6K).

### CITED1 Sequencing

**Figure 7:**
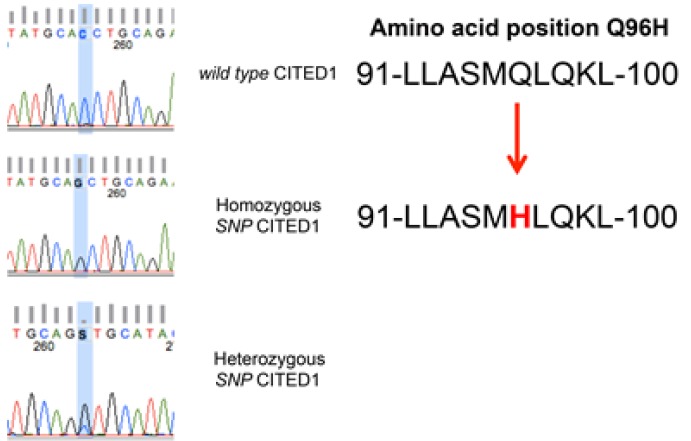
A homozygous single nucleotide polymorphism in the NES domain of *CITED1* (G864C; Q96H) was detected in 4/20 (20%) WT specimens sequenced Three of the 11 (27%) kidney specimens adjacent to a WT showed homozygous wild-type CITED1, 3 of 11 (27%) showed homozygosity for this CITED1 SNP, and the remaining 5 (46%) showed heterozygosity for this SNP. This SNP corresponds to amino acid region 91-100, which represents a CITED1 nuclear export signal (NES) motif.

To determine whether *CITED1* is mutated in clinical WT specimens to explain its aberrant subcellular localization, the *CITED1* gene locus was sequenced in 20 WT and in 11 kidney specimens having an adjacent WT. When compared with the wild-type CITED1 sequence reported by Shioda et. al [25], a homozygous single nucleotide polymorphism in the NES domain of *CITED1* (G864C; Q96H) was detected in 4/20 (20%) WT specimens sequenced (Figure 7). This SNP corresponds to amino acid region 91-100, which represents a CITED1 nuclear export signal (NES) motif [26]; however, the functional significance of this SNP was not examined in the current study. To validate specificity of this SNP, controls used for other experimental purposes in this study were sequenced similarly and showed wild-type CITED1 at this amino acid 96 (ie, wild-type WiT49 cells, pcDNA3-CITED1 plasmid DNA, pcDNA3-CITED1ΔNES plasmid DNA, WiT49 cells transfected with both wildtype and ΔNES CITED1 plasmids, two normal adult kidney specimens (Agilent and Clontech). Sequencing of pooled fetal kidney specimens (Clontech; number of kidneys not available) showed heterozygosity of this SNP. Interestingly, 3 of the 11 (27%) kidney specimens adjacent to a WT showed homozygous wild-type CITED1, 3 of 11 (27%) showed homozygosity for this CITED1 SNP, and the remaining 5 (46%) showed heterozygosity for this SNP. In summary, 8 of 11 (73%) kidney specimens adjacent to a WT showed evidence of this SNP, either as a heterozygous or homozygous event.

## DISCUSSION

These studies were designed to mimic the aberrant subcellular localization of CITED1 that is observed in WT blastema, its putative cancer stem cell. Because WT blastema shows robust expression of CITED1 in both the cytosol and nucleus, we established a model system that localizes CITED1 predominantly, but not exclusively, to either subcellular compartment in order to evaluate its potential mechanisms in each context. Interestingly, these studies reveal a dual role for CITED1 in WT, which indeed appears dependent on its differential subcellular localization. Specifically, when wild-type CITED1 was ectopically expressed in the cytosol, analogous to its subcellular localization in nephron progenitors, a pattern of gene expression emerged that suggests maintenance of stem cell function and inhibition of differentiation. When cytosolic CITED1 was overexpressed in the human WT cell line, WiT49, the foremost upregulated gene was *LGR5*, a marker of stem cells known to play a role in nephron formation and in the gastrointestinal tract [27]. Likewise, the foremost downregulated gene in this experiment was *CDH6*, which is a member of the cadherin gene family and is necessary for formation of the pretubular aggregate in the embryonic kidney, representing an early marker of epithelial differentiation during nephrogenesis. Along with a pattern of observed WNT inhibition both by examination of gene transcription (qPCR) and on TOP-FLASH reporter assay for β-catenin activity, these data together show that cytosolic CITED1 imparts a stem-like phenotype to WT, which may contribute to maintenance of a population of cancer stem cells. Moreover, upon ectopic expression of a *nuclear-enriched* CITED1 mutant (CITED1ΔNES), which remains transcriptionally active, WiT49 cells showed enhanced tumorigenicity, both in soft agar assay and in a xenograft model. The pattern of WNT inhibition both on TOP-FLASH assay and on examination of gene transcripts disappeared with expression of CITED1ΔNES, indicating that nuclear CITED1 may cooperate with β-catenin-dependent pathogenic responses in WT.

Ectopic expression of wild-type CITED1 in a human WT cell line foremost *induced LGR5* transcription, which was validated by immunohistochemistry in the resulting xenograft WiT49 tumors. A direct target of β-catenin, *LGR5* has been characterized through lineage tracing experiments as a stem cell marker in a variety of normal adult tissues that maintain high cellular turnover and are dependent on self-renewal properties, such as the intestinal crypt [28–30]. This feature of *LGR5*, to label normal multipotent stem cells, has been further extended to numerous malignancies [31–33]. Analogous to and consistent with our current observations, LGR5 overexpression in colon cancer cell lines was shown to repress WNT pathway genes and to enhance expression of epithelial to mesenchymal transition genes, which together promote a stem-like phenotype [34]. The adult kidney however does not harbor a known population of stem/progenitor cells poised to repopulate nephron epithelia after injury and has been shown to be without *LGR5* detection. A recent study characterizing *Lgr5* expression across murine kidney development showed its detection between e14 and postnatal day 7, after which its activity was no longer present [29]. LGR5 has recently been shown to label stem cells that play a role in nephron formation [27]. These authors assert that *Lgr5* confers stemness to cells of an early nephron structure, the S-shaped body, which was proposed to retain the capacity for self-renewal as well. In the current studies, *LGR5* mRNA was generally detected in greater content among the WT specimens, approaching the reference fetal kidney values, relative to the adjacent and reference adult kidney specimens.

In the same gene expression analysis of our experimental WT cell lines, we showed that the gene most significantly *repressed* by wild-type CITED1 was *CDH6*. CDH6 protein levels were reduced in cultures of both WiT49 experimental cell lines ectopically expressing either wild-type or CITED1ΔNES. Cadherin-6 (CDH6) in the embryonic kidney is required for the early pretubular aggregation of induced mesenchymal cells and subsequent epithelial conversion [35, 36]. After formation of the pretubular aggregate, the renal vesicle is the next earliest epithelial structure to appear in the nephrogenic zone and in which both CITED1 and SIX2 are completely absent [10] it is in this primitive nephronic element that CDH6 is initially detected [37]. Furthermore, an additional study recently identified CDH6 as a key downregulated gene in WT relative to the developmental kidney [38]. Putting together the observations that ectopic wild-type CITED1 expression in the WiT49 cell line foremost induces concomitant *LGR5 upregulation* and *CDH6 downregulation*, we speculate that this combined effect confers stem-like properties to the metanephric blastema and that WTs hijack this trait to maintain its putative cancer stem cell.

In keeping with this candidate mechanism of cytosolic CITED1 to impart a stem-like phenotype to WT, we examined whether this malignant context further exploits CITED1 developmental functions for the gain of unchecked self-perpetuation, analogous to a multipotent progenitor in embryonic tissues. In previous studies, we have shown that CITED1 represses canonical WNT/β-catenin signaling in kidney development, but its exact entry point into this complex pathway for such modification has not been clarified [9]. Canonical WNT/β-catenin activation in the nephrogenic zone of the developing kidney plays a role in the balance of nephron progenitor self-renewal versus differentiation and therefore may serve a gatekeeper role in regulating the CM to form the pretubular aggregate. Through specific interactions with either of two co-activators, CBP and p300, β-catenin directs a binary cell-fate decision to remain in a stem state (ie, CBP-dependent) or to differentiate (ie, p300-dependent) [39]. We speculate in our model that cytosolic CITED1 may influence β-catenin preferentially to interact with CBP and thereby confer perpetual stemness to WT blastema. Indeed, we have shown previously that CITED1 blocks WNT-dependent differentiation of cultured metanephric mesenchymes. In a human WT cell line, we now show that forced expression of wild-type CITED1 imparts a multi-level inhibition of the WNT pathway, appearing collectively and logically to maintain the stem state for this embryonal tumor. For example, *DKK1*, a known WNT antagonist, and its receptor, *KREMEN1*, are both upregulated in the present experimental WT model. *DKK1* specifically has been shown to inhibit nephrogenic progression in the developing kidney and to accumulate in both WT and hepatoblastoma [12, 40, 41]. In support of this pro-stem effect from the current studies, DKK1 and LGR5 appeared to be co-expressed in both non-cancerous kidney tissues and particularly in WT specimens. To exert its inhibitory effects on the WNT pathway, secreted DKK1 must bind to the WNT co-receptor, LRP6, which then forms a ternary complex with KREMEN1. As a result of this complexing, LRP6 is not available to bind WNT ligands and to form a ternary complex with Frizzled, which is a necessary event to activate canonical signaling [42]. Additionally, WNT2b, an effector of metanephric mesenchyme differentiation, is significantly *repressed* upon ectopic expression of wildtype CITED1 in WiT49 cells [18]. Several other WNT pathway mediators were observed to be up- or down-regulated in the context of ectopic CITED1 expression in WiT49 cells and moreover fit the logical pattern of repressing epithelial differentiation.

Because CITED1 shows context-dependent subcellular localization, with robust nuclear enrichment in malignant blastema, which again deviates from its cytosolic predominance in nephron progenitors of the embryonic kidney, we questioned what functional significance, if any, CITED1 might impart to WT pathogenesis. To answer this question, we developed and validated an experimental model that mimics the expression domain of CITED1 in clinical WT specimens. Foremost, when enriched experimentally in the nucleus of a WT cell line, CITED1ΔNES enhanced anchorage-independent growth, an assay that relies on cellular proliferation independent of extracellular controls. Although ectopic expression of wild-type CITED1 also showed marginally enhanced growth response in soft agar relative to control cells, it did not achieve the same magnitude of colony formation as did the mutant ΔNES. In cultured WiT49 cells, forced expression of wild-type and ΔNES CITED1 enhanced proliferation similarly, suggesting that nuclear enrichment confers an anchorage-independent growth advantage. Importantly, we validated these effects of forced nuclear CITED1 expression on proliferation and colony formation in a xenograft model, documenting similar pathogenic responses. Specifcally, CITED1ΔNES induced larger tumors having qualitatively more prevalent nuclear atypia. Taken together, these observations suggest that CITED1 nuclear enrichment may promote and speed WT cellular growth through additional mechanisms beyond proliferation alone.

One of the few WNT genes that was significantly altered in this ΔNES experimental line relative to the empty vector controls was *CTNNBIP1* (β-catenin-interacting-protein-1), which acts in the nucleus to bind and displace β-catenin (ie, CTNNB1) from TCF, thereby, inhibiting its transcriptional activity [43]. Speculatively, nuclear CITED1 may stabilize β-catenin oncogenic properties by repressing CTNNBIP1, a negative regulator of β-catenin-dependent transcription. Pertinent to WT, CTNNBIP1 is down-regulated in the context of loss of genetic material at 1p, and loss of heterozygosity at 1p in WT portends a higher risk for treatment failure [16]. Perhaps enrichment of CITED1 in the nucleus of WT blastema, which represses CTNNBIP1, may serve a similar oncogenic function.

Given the emerging theory from these studies, that CITED1 imparts a cancer stem cell phenotype to WT blastema having concomitant deregulation of cellular growth control, we supposed this model system could reveal opportunities to identify new and unique targets for therapeutic intervention. Interestingly, real time PCR screening of 84 cancer drug targets uncovered two genes that appeared significantly upregulated in response to CITED1 ectopic expression in a human WT cell line. First, *IGF2* (insulin-like growth factor 2) was significantly upregulated in the WiT49 cell line expressing wild-type CITED1. Notably, *IGF2* is positioned within the 11p15.5 locus (*WT2*) and is regulated through paternal genomic imprinting [44, 45]. Loss of imprinting of *IGF2* results in excessive production of this fetal growth factor, an event which has been identified in nearly 70% of WT and is associated with the overgrowth syndromes, Beckwith-Wiedemann and hemihypertrophy, that are well known to predispose to WT. Second, *PTGS2* (prostaglandin-endoperoxide synthase 2; protein product is COX2) was significantly upregulated in CITED1-expressing WiT49 cells on the gene expression microarray and was then validated on the real-time PCR cancer drug target array. COX2 has been shown in other cancer systems to promote tumor maintenance and disease progression, and in the developing kidney, COX2 metabolites have been reported to play important roles in early nephrogenesis and function [46, 47]. Importantly, *IGF2*, *PTGS2*, and *LGR5* are each targets of β-catenin. *IGF2* is a well-studied gene contributing to Wilms tumorigenesis but *PTGS2* has not been reported in the literature as playing a significant role in WT pathogenesis.

In summary, ectopic expression of CITED1 confers dual properties to a human WT cell line, depending on its subcellular localization. When enriched in the cytosol of WiT49 cells, CITED1 imparts a stem-like phenotype that may fuel perpetuity of a WT stem cell. When enriched in the nucleus, CITED1 appears to enhance pathogenic responses of this cultured and xenotransplanted WT cell line. CITED1 modification of WNT signaling may reveal targets to induce WT cancer stem cell differentiation and to repress β-catenin-driven oncogenicity. Additionally, clarification of CITED1 nucleo-cytosolic shuttling in WT is warranted.

## METHODS

### CITED1 constructs

To test the effects of differential subcellular localization in a human WT cell line, sequences for wildtype *CITED1* and a functional nuclear-enriched mutant were sub-cloned into the pcDNA3 expression vector (Life Technologies, Carlsbad, CA). Wild-type CITED1, tagged with the FLAG epitope at the amino terminus (pcDNA3-CITED1), is predominantly localized to the cytosol when transfected into human embryonic kidney cells (293-HEK cells) [26]. To mimic the CITED1 nuclear-enrichment observed in clinical WT specimens, point mutations in two amino acids (L165A and L167A) of the nuclear export signal (NES; substitutes alanine for leucine) of the FLAG-tagged human *CITED1* expression plasmid were created using a PCR-based site-directed mutagenesis kit (Agilent Technologies, Inc., Santa Clara, CA) and confirmed by direct sequencing. This mutant *CITED1* (pcDNA3-CITED1ΔNES) has been shown to retain its transcriptional activity and to be enriched in the nucleus of 293-HEK cells [26]. The pcDNA3 plasmid (control: empty vector only) contains a neomycin resistance cassette for pressure selection of transfected cells using Geneticin (G418; Life Technologies).

### Cell lines

The human WT cell line, WiT49, was used for all CITED1 misexpression studies [48]. To generate stable experimental CITED1 WT cell lines, WiT49 cells were transfected with 24μg plasmid and Lipofectamine 2000 (Life Technologies). Transfected cells were selected by adding 1 mg/mL of Geneticin (G418) to the media for five weeks, at which time protein was extracted from cell lysates to assay for FLAG-tagged CITED1. Biologic replicates were defined as a different culture passage after stable transfection had been validated and not to exceed passage 9. MCF7 cells (American Type Culture Collection; Manassas, VA) were used as a CITED1 positive control for Western blotting. Human embryonic kidney (HEK) 293 cells and Cos cells (ATCC) were used as additional control lines.

### Western blot

CITED1 protein content was estimated by Western blot, as previously described [7, 10]. Immunoblots were performed using affinity-purified rabbit anti-CITED1 (1:1000 dilution; Lab Vision Corp), M2 anti-FLAG antibody (1:1000 dilution; Sigma Chemical Co, St Louis, MO), and mouse anti-β-actin (1:5000 dilution; Sigma). Densitometry was performed using Photoshop (Adobe, San Jose, CA). Cadherin-6 (CDH6) Western blots were performed similarly using rabbit anti-β-CDH6 antibody (1:1000 dilution; Cell Signaling Technology, Beverly, MA). Results were normalized to β-actin optical densities. For embryonic sources of CITED1 and CDH6, murine fetal kidneys (MFK; e18.5) specimens were used [10].

### *In vitro* and *in vivo* tumorigenesis assays

#### In vitro

To evaluate the effects of CITED1 misexpression on tumorigenicity, cells were placed in soft agar to test anchorage-independent growth. Briefly, 60,000 cells were plated in 0.4% SeaPlaque top agar (Lonza, Walkersville, MD) and layered on 0.8% SeaPlaque agar in a 10 cm plate. Plates were incubated for 3 weeks at 37°C in 5% CO_2_ and then stained with 10mg/mL MTT (Sigma, St. Louis, MO) for 1 hour at 37°C. Colonies were counted using a GelCount instrument (Oxford Optronix, Oxford, United Kingdom). Four biological replicates were performed for each experimental cell line.

To evaluate the effect of CITED1 nuclear enrichment on WiT49 proliferation, the CellTiter96 non-radioactive kit was used (Promega, Madison, WI). Briefly, 5,000 cells were plated in a 96-well flat bottom plate in media containing 0.2% FBS. Cells were grown for 1, 24, 48 and 72 hours, and 15uL dye reagent was added to each well for four hours. Plates were read in a SpectaMax spectrophotometer (Molecular Devices, Sunnyvale, CA) at 570nm and 650nm, and final results were calculated by subtracting the 650nm from the 570nm values.

#### In vivo

Forty-five female SCID-Beige mice at 4-6 weeks of age were obtained from Charles River Laboratories (Wilmington, MA). Each of the three experimental WiT49 cell lines was injected into the right flank of 15 immunodeficient mice at a concentration of 2×10^6^ cells suspended in 250mL of growth factor-depleted Matrigel (BD Biosciences, Franklin Lakes, NJ). Necropsy was performed 10 weeks post-injection. Resulting tumors were weighed in grams, and tumor volumes were estimated in mm^3^ using digital calipers (VWR, Suwanee, GA) and the formula for a prolate ellipsoid (0.52 × length × width × height), as described [13, 49].

### Immunohistochemistry

#### *In vitro* immunofluorescence

CITED1 transgene expression was verified in 293-HEK cells and each experimental WiT49 cell line using *in vitro* immunofluorescence, as described [50]. CITED1 was probed with affinity-purified rabbit anti-CITED1 (1:50 dilution; Lab Vision Corp., Fremont, CA) and visualized using goat anti-rabbit FITC-labeled antibody (1:50; Jackson ImmunoResearch, West Grove, PA).

#### Experimental Wilms tumors

To characterize expression of CITED1, NCAM, and LGR5 in experimental WiT49 tumors, we performed immunoperoxidase staining of formalin-fixed, paraffin-embedded tissue sections, as described [10]. Sections were incubated in rabbit anti-CITED1 (1:50 dilution), rabbit anti-NCAM (1:100 dilution; Cell Signaling, Danvers, MA), and rabbit anti-LGR5 (1:20; ABGENT, San Diego, CA). Goat-anti-rabbit secondary antibody (1:200 dilution; Santa Cruz Biotechnology, Santa Cruz, CA) was applied to tissues at room temperature for 45 minutes. Tissues were visualized with an anti-rabbit Dako EnVision+ HRP/DAB System (DakoCytomation; Carpinteria, California).

### Gene Expression Microarray

To evaluate the effects of ectopic CITED1 expression on WiT49 gene expression profiles, microarray experiments were performed using the HumanGene 1.0 ST (Affymetrix, Santa Clara, CA). Total RNA was isolated from three biological replicates (passages 3, 5, and 7) of each experimental cell line and analyzed according to protocol [10].

### WNT pathway and Cancer Drug Targets RT^2^ Profiler™ qPCR Arrays

To evaluate the effects of CITED1 misexpression on the WNT pathway and to reveal candidate drug targets in this WT model, Qiagen qPCR arrays were obtained for the human WNT Signaling Pathway (PAHS-043) and the human Cancer Drug Targets (PAHS-507). Briefly, 1μg of RNA was reverse transcribed using the RT^2^ First Strand Kit (Qiagen, Valencia, CA). Reactions were performed using this cDNA and RT^2^ SYBR Green master mix according to the manufacturer's instructions (Qiagen). Four biological replicates (passages 3, 4, 5, and 6) were used for all analyses except passages 3-5 of the ΔNES cells were used for the Cancer Drug Targets.

### Validation studies in human Wilms tumor and adjacent kidney specimens

#### Clinical tissues

To validate genes of interest identified in the microarray expression analyses, real-time PCR was performed on fresh WT and adjacent kidney specimens, obtained immediately after nephrectomy (IRB approval #100734 and #020888) and archived in our laboratory tissue repository. Total RNA was isolated from snap frozen tissue of 20 consecutive treatment-naive WTs and 12 adjacent kidney specimens using RNAzol (Tel-Test Inc., Friendswood, TX) and purified using RNeasy mini kit (Qiagen, Valencia, CA). 3μg of RNA were reverse transcribed using Superscript II (Life Technologies) and oligo(dT) primers (Applied Biosystems). Quantitative real-time PCR was performed using iQ SYBR Green Master Mix (Bio-Rad, Hercules, CA) on a Bio-Rad iCycler. For additional controls, we obtained pooled RNA isolated from two normal adult kidneys and from fetal kidneys (gestational age 12 – 30 weeks).

#### Normal adult and fetal kidney RNA

The cellular biology of ipsilateral kidney tissue arising adjacent to a WT may not be truly normal, due to cancer field effects and/or to physical compression and relative ischemia from the often-massive size of these lesions. As a result, to provide reference for gene activity in otherwise “normal” kidney tissue, we obtained adult and fetal kidney total RNA (n=2 samples per group, 1 each from: Clontech, Mt. View, CA and Agilent Technologies, Santa Clara, CA). RNA preparation and quantitative PCR was performed as above.

Real-time PCR results for each GOI were normalized to the mRNA content of the housekeeping gene, *GAPDH*, for all conditions. Primer sequences for PCR are available on request.

### Canonical WNT reporter assay

To determine the biological relevance of CITED1 subcellular localization on activation of the canonical WNT signaling pathway, we performed a TOP-FLASH luciferase assay for β-catenin activity. Each WiT49 cell line was transfected with ExtremeGene HP (Roche Applied Sciences, Indianapolis, IN) using a 3:1 ratio according to the manufacturer's instructions in 6-well plates for 4 different passages with the TOP-FLASH and FOP-FLASH plasmids (gift of Ethan Lee, Vanderbilt). After 24 hours of transfection, the media was removed, and WNT3a conditioned media mixed in a 1:1 ratio with normal growth media was placed on the cells. The WNT3a conditioned media was left on the cells for 24 hours, and the luciferase assay was performed at this time using a luciferase assay kit (Promega, Madison, WI) according the manufacturer's instructions. In addition, HEK293 STF cells were transfected with each of the three plasmids to determine effects of CITED1 in a developmental system.

### CITED1 sequencing in clinical Wilms tumor

To determine whether mutations in the nuclear export signal of CITED1 are present in clinical WT specimens or adjacent normal kidney tissue, we isolated and purified total RNA from snap-frozen tissues using RNAzol (Tel-Test Inc, Friendswood, TX) and RNeasy Mini Kits (Qiagen, Germantown, MD). Isolated RNA was quantified using a SpectraMax M5 UV spectrophotometer (Molecular Devices, Sunnyvale, CA). Reverse transcription (RT) of 3 μg of RNA was performed using Superscript II reverse transcriptase (Invitrogen, Grand Island, NY) and oligodT primers (Applied Biosystems, Foster City, CA) to synthesize cDNA suitable for direct sequencing. PCR amplification of the human *CITED1* gene using Taq Polymerase was performed on 20 WT specimens and adjacent areas of normal kidney. PCR primers were as follows: *CITED1* forward 5′-ATG CCA ACA ACG TCG AGG CC-3′ and *CITED1* reverse 5′-TTA GCA GCT AGA TGG AAA GT-3′. PCR conditions consisted of 1X PCR buffer, 400nM of each primer, 200mM dNTP's (Promega, Madison, WI), and 0.05 units/mL Taq polymerase (Sigma, St. Louis, MO) with the following cycling conditions: a) 1 cycle of 95°C for 10 minutes, b) 35 cycles of 95°C for 30 seconds, 55°C for 30 seconds, and 72°C for 30 seconds, and c) 1 cycle of 72°C for 10 minutes. PCR products were gel purified using a QIAquick Gel Extraction Kit (Qiagen, Valencia, CA) and subsequently directly sequenced by GeneHunter Corporation (Nashville, TN). Generated sequences and chromatographs were compared to wild-type *CITED1* using the Basic Local Alignment Search Tool (BLAST; NCBI, Bethesda, MD) to determine the mutational status of each specimen.

### Statistics

Three pair-wise comparisons of normalized colony counts were performed using the Wilcoxon signed-rank test. A linear mixed effects model was used to test for different baseline numbers of proliferate cells, as well as different increases in proliferate cells over time. Differences in the distributions of tumor volumes and weights were compared using standard analysis of variance (ANOVA). Individual means were compared using Tukey's honestly significant difference to control the family-wise type I error rate at 5% (e.g., control for multiple comparisons).

Microarray data were normalized using the Robust MultiChip Averaging algorithm as implemented in the Bioconductor package *Affy*. The gene expression values were transformed to log2 scale. The linear model implemented in the Bioconductor *LIMMA* package was used, and contrasts between CITED1 and pcDNA3, NES and pcDNA3, and CITED1 and NES were performed to detect differentially expressed probe sets. This model has the same interpretation as the standard one-way analysis of variance (ANOVA). To control for false discovery rates, *p* values for moderated *t* tests were adjusted using the method of Benjamini and Hochberg. Data from the real-time PCR arrays (deltaCt values) among the 3 groups were compared using ANOVA. To compare the content of microarray “genes of interest” between WTs and kidney specimens, a nonparametric test of the deltaCT values was used (Anderson-Darling Test).

To compare relative content of genes of interest in WT and kidney specimens (adjacent kidneys only), the Wilcoxon rank sum test was applied and box plots generated; pooled adult and fetal values served as reference points only and are also shown. To determine any association either positive or negative between genes of interest in non-cancerous kidneys (i.e., pooled adult and fetal, and adjacent specimens), Spearman correlation coefficients were determined; the same was performed for WT specimens as well.
